# Phenotypic and genotypic characterization of *Enterococcus cecorum* strains associated with infections in poultry

**DOI:** 10.1186/s12917-016-0761-1

**Published:** 2016-06-27

**Authors:** Beata Dolka, Dorota Chrobak-Chmiel, László Makrai, Piotr Szeleszczuk

**Affiliations:** Department of Pathology and Veterinary Diagnostics, Faculty of Veterinary Medicine, Warsaw University of Life Sciences-SGGW, Nowoursynowska 159c St., Warsaw, 02-776 Poland; Department of Preclinical Sciences, Faculty of Veterinary Medicine, Warsaw University of Life Sciences-SGGW, Ciszewskiego 8 St., Warsaw, 02-786 Poland; Department of Microbiology and Infectious Diseases, Faculty of Veterinary Science, Szent István University, Hungária krt. 23-25, Budapest, H-1143 Hungary

**Keywords:** *Enterococcus cecorum*, Phenotyping, Genotyping, PFGE, Enterococcal spondylitis, Chicken

## Abstract

**Background:**

From the beginning of the 21^st^ century *Enterococcus cecorum* has emerged as a significant health problem for poultry raised under intensive production systems. To obtain new insights into this bacterial species, we investigated 82 clinical isolates originating from different poultry flocks in Poland between 2011 and 2014.

**Results:**

Phenotypically, isolates from clinical cases showed ability to growth at low temperatures (4 °C, 10 °C), and differences in growth at 45 °C (74.4 %). Survival at high temperatures (60 °C, 70 °C) was observed for 15, 30 min. More than half of strains survived at 60 °C even after prolonged incubation (1 h), but none survived after 1 h at 70 °C. Total growth inhibition was observed on agar supplemented with tergitol or potassium tellurite. Relatively high number of isolates gave positive reactions for β-galactosidase (βGAL 80 %), Voges Proskauer test (60 %), less for β-mannosidase (17 %), glycogen and mannitol (12 %). The metabolic fingerprinting for *E. cecorum* obtained in Biolog system revealed ability to metabolise 22 carbon sources. Only 27/82 strains contained ≥ 1 virulence genes of tested 7, however 2.4 % isolates carried 6. Increased antimicrobial resistance was observed to enrofloxacin (87 %), teicoplanin (85 %), doxycycline (83 %), erythromycin (46 %). Most strains (75/82) showed multidrug resistance. The single isolate was resistant to vancomycin (VRE) and high level gentamicin (HLGR). Linezolid resistance among clinical isolates was not found. PFGE revealed diversity of *E. cecorum* from cases. It could be assumed that transmission of pathogenic strains between flocks regardless of type of production or geographical region may be possible.

**Conclusions:**

Clinical infections in poultry caused by *E. cecorum* may indicated on new properties of this bacterial species, previously known as a commensal. Despite many common phenotypic features, differences were found among clinical isolates. Several, widely distributed pathogenic *E. cecorum* strains seemed to be responsible for infection cases found in different poultry types.

**Electronic supplementary material:**

The online version of this article (doi:10.1186/s12917-016-0761-1) contains supplementary material, which is available to authorized users.

## Background

First time *Enterococcus cecorum* was isolated from cecal flora of chickens and described as *Streptococcus cecorum* in 1983, thereafter well known as commensal in gastrointestinal tract of various mammals and birds [1]. On the other hand, *Enterococcus cecorum* belongs to opportunistic pathogens and may also play a role as etiological agent of diseases in humans (nosocomial infections) [2, 3], chickens [4], and racing pigeons [5]. Recently, this bacteria appears to be a new threat (“emerging pathogen”) to poultry industry worldwide [6–15]. *E. cecorum* has been increasingly recognized as a cause of enterococcal spondylitis (ES), previously called enterococcal vertebral osteoarthritis (EVOA) in chickens [12]. Disease outbreaks were diagnosed mostly in broiler chicken flocks raised under an intensive production system. Clinically affected birds suffered from locomotor problems due to compression of the spinal cord at the thoracic vertebrae resulting from *E. cecorum* induced osteomyelitis and due to femoral head necrosis (FHN) [6, 7, 9, 12, 13]. Disease outbreaks can lead to high morbidity, mortality, culling, carcass condemnations, and may result in severe economic losses within a short time [9]. Recently, poultry or domestic animals (cats, dogs) are thought to be a possible source for transmission leading to *E. cecorum–*associated septicaemia in humans [2, 3].

Various methods using conventional biochemical tests and molecular techniques have been commonly used for identification and typing enterococci [16–18]. Pulsed field gel electrophoresis (PFGE) is considered to be the “gold standard” for subtyping enterococci and has been used extensively for molecular epidemiological characterization of enterococcal outbreaks [19, 20]. The PCR assay based on specific amplification followed by sequencing and nucleotide sequence comparison of target genes (such as 16S ribosomal RNA, *sod*A, *ddl*, *tuf*, *gro*ESL) or tDNA-PCR have served for the genotypic identification of enterococci [21–23].

Despite of available literature biochemical and molecular analysis of *E. cecorum* strains with poultry origin isolated in Europe are still limited. Moreover, there is not enough data regarding the properties of isolates, usually referred as pathogenic for poultry [1, 7, 8, 10]. The purpose of this study was phenotypic characterization of clinical *E. cecorum* isolates associated with infections in poultry and investigation their genetic relatedness.

## Methods

### Bacterial isolates

Eighty two *E. cecorum* isolates of poultry-origin used in this study were obtained from archival bacterial collection deposited at Department of Pathology and Veterinary Diagnostics, or were obtained from clinical specimens submitted by veterinarians for routine diagnostic work to the Diagnostic Laboratory in Division of Avian Diseases, Faculty of Veterinary Medicine at the Warsaw University of Life Sciences-SGGW (Poland). Authors ensure that the ARRIVE guidelines were followed. Among 82 clinical strains collected between 2011 and 2014, 49 came from broiler chicken flocks (CB), 20 from broiler breeder flocks (BB), 10 from commercial layer flocks (CL), 2 from geese flocks (G) and 1 from turkey flock (T). According to adopted criteria in this study, one *E. cecorum* isolate represented one different flock in which clinical problems due to *E. cecorum* infection were reported by veterinarians on farms. Affected birds displayed a variety of clinical signs, however in all types of flocks the lameness, paralysis, hock sitting, weakness, pododermatitis, decreased water and food intake were usually noted. Subsequently*,* disease caused lower results of production, increased losses due to mortality and culling. Necropsies and pathological examinations revealed usually femoral head necrosis, (purulent) arthritis*,* fibrinous pericarditis, endocarditis, hepatitis and congested lungs. Characteristic osteomyelitis lesions at caudal thoracic vertebrae we found only in chicken flocks (mainly in CB). Isolates were recovered from tissue samples such as vertebral column, femoral heads, heart, liver, lungs or yolk sac, which were collected during necropsy.

### Bacterial analysis

The tissue samples were inoculated onto Columbia agar with 5 % sheep blood (CA) (Graso, Poland) and agar plates with esculin (KAA, Biocorp, Poland; Enterococcosel Agar, Graso, Poland), then incubated at 37 °C for 24 h in a CO_2_-enriched atmosphere*.* Bacteria were identified as *Enterococcus* based on their phenotypic properties such as colonial morphology, hemolysis (on CA), Gram-staining, catalase production (using a 3 % H_2_O_2_), cytochrome oxidase production (OXItest, Erba Lachema s.r.o., Czech Republik), and esculin hydrolysis (Enterococcosel Agar, KAA). Pigment production was visually assayed by growing the bacteria on CA for 24 h and scraping off the growth with a white cotton swab. Motility was examined using Motility Test Agar (Graso, Poland). The ability to growth was estimated in 6.5 % NaCl (salt tolerance test) after 48 h at 37 °C, and on different media (Graso, Poland) (Table 2). Serological identification of Lancefield group was conducted by rapid latex agglutination method using Slidex Strepto Plus D (bioMérieux, France). Tests for *E. cecorum* growth were performed in BHI broth (Brain-heart infusion; bioMérieux, France) tubes preincubated at 4 °C, 10 °C, 45 °C for 24 h. Then cultures in BHI broth were spread onto CA and incubated at 37 °C. The growth response was assessed after 24 h and 48 h. The ability to survive at 60 °C, 70 °C was estimated for 15 min, 30 min, 1 h in BHI broth tubes, followed by incubation of inoculated CA plates. The results were recorded after 24 h and 48 h.

### Biochemical tests

Identification to the species level based on biochemical characterization was performed by API rapid ID 32 STREP (bioMérieux, France) and on the basis of carbon source utilisation using Biolog system (Biolog Inc., Hayward, USA)*.* Isolates (*n* = 13) were determined according to Biolog GP2 MicroPlates, which performed 95 discrete tests simultaneously and gave a characteristic reaction pattern (metabolic fingerprint). The MicroPlates were incubated at 37 °C and read visually after 4 h and 24 h. The metabolic fingerprint patterns were compared and identified using the MicroLog™ 4.20.05 database software.

### Virulence factors

All 82 isolates were tested for the presence of seven virulence factors: *asa1* (aggregation substance), *gelE* (gelatinase), *hyl* (hyaluronidase), *esp* (enterococcal surface protein), *cylA* (cytolisin), *efaA* (endocarditis antigen), *ace* (collagen-binding protein) according to Martín-Platero et al. [24], Jung et al. [5] using duplex PCRs (*asa1/gelE, cylA/esp, efaA/ace*) and single PCR (*hyl*). PCR reaction mix contained 12.5 μl DreamTaq PCR Master Mix (Thermo Fisher Scientific Inc., USA) 0.3 μl of each primer (50 pmol/μl), 4 μl DNA and PCR-clean water (added up to a volume of 25 μl). Thermocycler conditions were as follows: initial denaturation at 94 °C for 5 min, followed by 30 cycles: denaturation at 94 °C for 1 min, annealing at 56 °C for 1 min (55 °C for *efaA*/*ace*), extension at 72 °C for 1 min, followed by final extension step 72 °C for 10 min and a 4 °C hold. Amplification products (10 μl) were analyzed by 1.2 % agarose gel electrophoresis after ethidium bromide staining and visualized under UV light (UVP, USA). A 100-bp DNA ladder (Thermo Fisher Scientific Inc., USA) was used as a molecular size marker.

Production of gelatinase was additionally determined using Difco Nutrient Gelatin (BD, USA) according to the manufacturer’s recommendations. The tubes inoculated with *E. cecorum* ATCC 43198, *S. aureus* ATCC 25923 (gelatinase positive), *E. coli* ATCC 25922 (gelatinase negative) and an uninoculated tube were used for quality control testing.

### Antibiotic susceptibility

Susceptibility for 13 antimicrobial agents: amoxicillin/clavulanic acid (AUG 20/10 μg), ampicillin (AP 10 μg), penicillin (PG 10 μg), enrofloxacin (ENF 5 μg) tetracycline (TEC 30 μg), nitrofurantoin (NI 300 μg), doxycycline (DXT 30 μg), chloramphenicol (C 30 μg), erythromycin (E 15 μg), teicoplanin (T 30 μg), vancomycin (VA 30 μg), high level gentamicin (GM 120 μg) and linezolid (LZD 30 μg) was tested by Kirby-Bauer disk diffusion method and the results were interpreted according to Clinical and Laboratory Standards Institute guidelines [25]. The criteria for selection of antibiotics based on CLSI guidelines for *Enterococcus* spp. and on their practical significance for the clinical use. Among tested antibiotics, tetracycline, doxycycline, amoxicillin, enrofloxacin have been actually approved for use in poultry (erythromycin until 2014) and have practical relevance. Vancomycin resistance genes (*van*A, *van*B) were tested by PCR using primers and conditions previously reported [24]. *Staphylococcus aureus* ATCC 25923 (vancomycin susceptible), *E. faecalis* ATCC 51299 (vancomycin resistant), *E. cecorum* ATCC 43198 were used as controls.

### Molecular identification

Rapid extraction of bacterial genomic DNA was carried out by using boiling method. PCR assay targeting *sod*A gene was performed for identification and determination the diversity of 82 *E. cecorum* strains [22]. PCR products were visualized after electrophoresis on agarose gel (2 %) by staining with ethidium bromide, then purified using GeneMATRIX PCR/DNA Clean-Up Purification Kit (EURx, Poland) and submitted for sequencing to commercial services (IBB PAN, Genomed, Poland). The *sod*A gene sequences were analyzed with NCBI BLAST. The genetic distance**s** based on the partial sequences of *sod*A was calculated by the two-parameter method of Kimura by using the MEGA6, and the phylogenetic tree was constructed using the Neighbor-Joining method (NJ) with 1000 bootstrap replicates.

### PFGE

The standard PFGE procedure was adapted from previously published studies with minor modifications [18, 26, 27]. The 82 *E. cecorum* strains were cultured overnight on CA and then suspended in sterile saline to obtain the density of 3.5 on McFarland scale and centrifuged 10 min. at 4000 rpm/min. The bacterial pellets were mixed with 150 μl Tris-EDTA buffer solution (10 mM Tris-HCl, 1 mM disodium EDTA, pH 8.0) and 150 μl liquid 2 % agarose (InCert Agarose, Lonza, Rockland, USA) and small discs were formed (20 μl). The solidified discs were incubated at 37 °C for 18 h in 1 ml of EC buffer (6 mM Tris-HCl pH 8.0, 1 M NaCl, 0.1 M EDTA, 0.2 % deoxycholate, 0.2 % sarkosyl) containing 10 mg lysozyme (A&A Biotechnology, Poland), and 0.02 mg RNase A (Thermo Fisher Scientific Inc., USA). DNA discs were washed 3 times in 5 ml EBS solution (0.5 M EDTA pH 9.0, 1 % sarkosyl) and incubated overnight at 50 °C in 1 ml EBS solution containing 1 mg of proteinase K (ESP buffer) (A&A Biotechnology, Poland). Then the discs were washed 4 times (each time upside down for 30 times at room temperature) with 10 ml TE buffer (10 mM Tris, 1 mM EDTA, pH 8.0) and stored in 1 ml TE buffer at 4 °C. Subsequently, each disc was pre-incubated in 100 μl restriction buffer for 30 min at room temperature. The agarose discs were digested with *Sma*I (20 U/μl; Fermentas, Lithuania) overnight (at 37 °C). The restriction fragments were separated by clamped homogenous electric field (CHEF) electrophoresis with a CHEF-DR II System (Bio-Rad Laboratories, USA) in a 1.2 % (w/v) agarose gel using pulse time at 0.5 s followed by 35 s at 6 V/cm and temperature 14 °C for 24 h [17]. Afterwards the gel was stained with ethidium bromide for 30 min, then washed in distilled water for 30 min, photographed under UV light and documented in the system VersaDoc (Bio-Rad Laboratories, USA). Lambda Ladder PFG marker (New England Biolabs Inc., USA) was used as molecular size marker. Gel images were analyzed by Gel Compar II version 6.6 (Applied Maths, Belgium) and cluster analysis was performed by UPGMA using dice similarity coefficient with optimization set at 1 % and position tolerance at 1 %. Isolates were clustered using an 80 % homology cut-off, above which strains were considered to be closely related and assigned to the same PFGE type [19].

## Results

### Phenotypic characterization

Table 1 shows results of conventional tests and effects of different temperatures on the growth and survival of *E. cecorum* strains. Bacterial growth was characterized on 7 different microbiological media (Table 2).

**Table 1:** Test or characteristic for *E. cecorum* isolates (*n* = 82)

Test or characteristic	*E. cecorum* isolates from clinical cases
Hemolysis	α (strong)
Gram-staining	Gram-positive
Cell morphology	ovoid cocci (single, double or short chains)
Catalase-production	negative
Oxidase-production	negative
Yellow pigment-production	negative
Lancefield group D	negative
Motility	negative
Halotolerance (6.5 % NaCl)	limited growth
Growth at:	% positive (n)
4 °C	100 % (82)
10 °C	98.8 % (81)
45 °C	74.4 % (61)
Survival at 60 °C for:	% positive (n)
15 min	76.8 % (63)
30 min	64.6 % (53)
1 h	54.9 % (45)
Survival at 70 °C for:	% positive (n)
15 min	36.6 % (30)
30 min	15.9 % (13)
1 h	0 % (0)

**Table 2:** Results of *E. cecorum* (*n* = 82) growth on different media

Medium	Observed growth (YES/NO)	Description of colonies of *Enterococcus cecorum*
Columbia Agar with 5 % Sheep Blood (CA)	YES	Small, round, white-grey colonies with α-hemolysis
Columbia CNA Agar with 5 % Sheep Blood	YES	Small, grayish colonies with α-hemolysis, resistant to two antibiotics colistin and nalidixic acid
Edwards Agar with 5 % sheep blood	YES	Blue-grayish coloured colonies with α-hemolysis
Bile Esculin Azide Agar (Enterococcosel Agar)	YES	Colonies beige with strong black halos
KAA agar (Kanamycine Esculin Azide Agar)	YES (weak)	Brown to black colonies and blackening zones around the colonies
Slanetz and Bartley Agar (with tetrazolium chloride)	NO or poor	Red, maroon colonies
TCC agar with tergitol	NO	Total inhibition
Tellurite Agar (potassium tellurite)	NO	Total inhibition

### Biochemical tests

The strains were identified as *E. cecorum* with the API rapid ID 32 STREP and Biolog system. API revealed perfect identification profile (ID 99.9 %, T 0.83) for 40 (49 %) *E. cecorum* strains, very good identification (ID 99.9 %, T 0.67) for 21 (26 %) strains, good identification (ID 99.8 %, T 0.38) for 2 (2 %) strains, doubtful profile (99.9 %, T 0.4) for 16 (20 %) strains, and unacceptable profile for 3 (4 %) strains. Among perfect identification profiles for *E. cecorum*, the code 6717–4607–131 was recorded the most often. Based on the analysis of 82 obtained profiles in API (each with 32 tests), we defined one common code 2317–4607–111 for clinical strains which gives perfect identification as *E. cecorum* with the API database. Biochemical results obtained in API were presented in Table 3. The vast majority of isolates was positive in tests for βGLU, RAF, SAC, MβDG, CDEX (100 %), αGAL, RIB, TRE (99 %), MAL, MEL (98 %). All isolates were completely negative for ADH, APPA, HIP, PYRA, LARA. The discrepancies among tested and control isolates or recommendations for *E. cecorum* were noted in 6 tests: βGAR, MAN, VP, βGAL, GLYG, βMAN.

**Table 3:** Percent of positive reactions (%) in rapid ID 32 STREP (bioMérieux, France) for clinical *E. cecorum* isolates in this study (*n* = 82) compared with standard isolates (manufacturers recommendations) and control strain (*E. cecorum* ATCC 43198)

Parameter	% Positive reactions in rapid ID 32 STREP for *Enterococcus cecorum*		
	Clinical isolates (this study) % (n)	Manufacturers recommendations %	Reference strain *E. cecorum* ATCC 43198 (+/-)
ADH	0 (0)	0	-
βGLU	100 (82)	100	+
βGAR	73 (60)	11	-
βGUR	94 (77)	88	+
αGAL	99 (81)	100	+
PAL	71 (58)	94	+
RIB	99 (81)	98	+
MAN	12 (10)	38	-
SOR	10 (8)	11	-
LAC	88 (72)	100	+
TRE	99 (81)	100	+
RAF	100 (82)	88	+
VP	60 (49)	66	-
APPA	0 (0)	0	-
βGAL	80 (66)	33	-
PYRA	0 (0)	0	-
βNAG	82 (67)	88	+
GTA	89 (73)	94	+
HIP	0 (0)	1	-
GLYG	12 (10)	27	-
PUL	4 (3)	0	-
MAL	98 (80)	100	+
MEL	98 (80)	98	+
MLZ	88 (72)	55	+
SAC	100 (82)	100	+
LARA	0 (0)	0	-
DARL	1 (1)	0	-
MβDG	100 (82)	98	+
TAG	65 (53)	64	+
βMAN	17 (14)	41	-
CDEX	100 (82)	66	+
URE	6 (5)	0	-

ADH (arginine dihydrolase), βGLU (β-glucosidase), βGAR (β-galactosidase), βGUR (β-glucuronidase), αGAL (α-galactosidase), PAL (alkaline phosphatase), RIB (ribose), MAN (mannitol), SOR (sorbitol), LAC (lactose), TRE (trehalose), RAF (rafinose), VP (Voges Proskauer, aceton production), APPA (alanyl-phenylalanyl-proline arylamidase), βGAL (β-galactosidase), PYRA (pyroglutamic acid arylamidase), βNAG (N-acetyl-β-glucosaminidase), GTA (glycyl-tryptophan arylamidase), HIP (hydrolysis of hipurate), GLYG (glycogen), PUL (pullulane), MAL (maltose), MEL (melibiose), MLZ (melezitose), SAC (saccharose), LARA (L-arabinose), DARL (D-arabitol), CDEX (cyclodextrin), MβDG (methyl-βD-glucopyranoside), TAG (tagatose), βMAN (β-mannosidase), URE (urease)

**Fig. 1:**
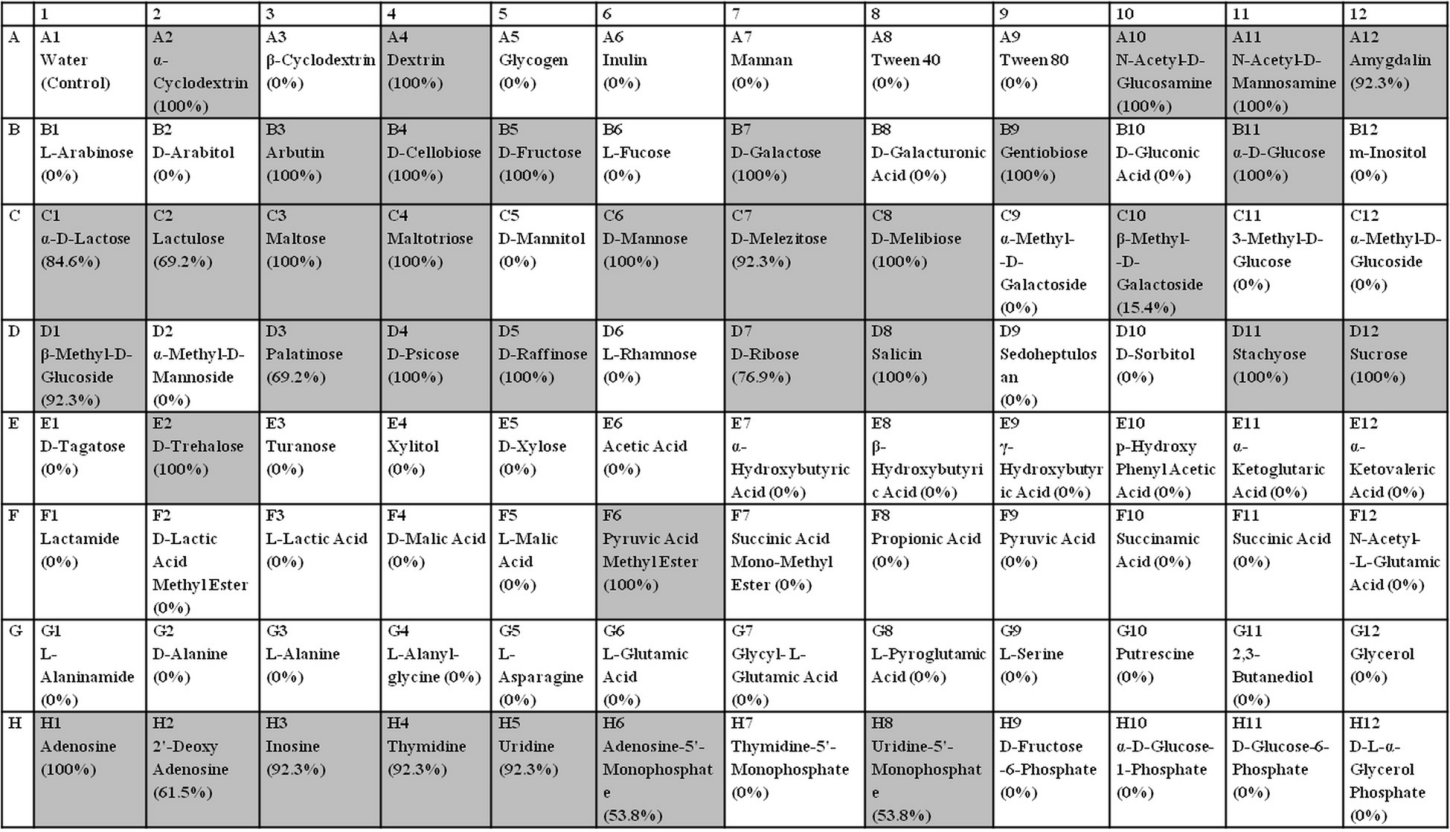
Percent of positive profiles for *Enterococcus cecorum* in Biolog GP2 MicroPlate™

All of examined isolates were identified as *E. cecorum* in Biolog system (index: probability 91.7 %, similarity 0.806). The metabolic fingerprinting for *E. cecorum* was showed in Fig. 1. All of the examined isolates were able to metabolise 22 carbon sources (α-cyclodextrin, dextrin, N-acetyl-D-glucosamine, N-acetyl-D-mannosamine, arbutin, D-cellobiose, D-fructose, D-galactose, gentiobiose, α-D-glucose, maltose, maltotriose, D-mannose, D-melibiose, D-psicose, D-raffinose, salicin, stachyose, sucrose, D-trehalose, pyruvic acid methyl ester, adenosine). Not all of examined isolates were able to metabolise 14 carbon sources: amygdalin, D-melezitose, β-methyl-D-glucoside, inosine, thymidine, uridine (metabolised by 92.3 % strains), α-D-lactose (84.6 %), D-ribose (76.9 %), lactulose, palatinose (69.2 %), 2’-deoxy-adenosine (61.5 %), adenosine-5’-monophosphate, uridine-5’-monophosphate (53.8 %), β-methyl-D-galactoside (15.4 %). Further 59 carbon sources present in the GP2 microplate were not utilised by *E. cecorum*.

### Virulence factors

Of all 82 *E. cecorum* strains, 22 (26.8 %) were positive for *asa1*, 21 (25.6 %) for *gelE*, 12 (14.6 %) for *ace*, 11 (13.4 %) for *efaA*. The *cylA* and *esp* PCR amplification yielded positive results in 4 (4.9 %) and 2 (2.4 %) *E. cecorum* strains. The *hyl* gene was not detected in any strain. The isolates from CB were positive for *asa1* (24.5 %), *gelE* (22.4 %), *ace* (14.3 %), *efaA* (14.3 %), *cylA* (2.1 %). The isolates from BB were positive for *asa1* (20 %), *gelE* (20 %), *ace* (15 %), *esp* (10 %)*, cylA* (10 %). The isolates from CL were positive for *asa1* (60 %)*, gelE* (60 %), *efaA* (20 %), ace (20 %). None of 7 virulence factors was found in isolates from G and T flocks. Most of virulence-gene positive isolates (11; 13.4 %) contained 2 of tested 7 virulence genes, then 6 (7.3 %) *E. cecorum* contained 4 virulence genes, 5 (6.1 %) harbored 1 virulence gene, while 3 (3.7 %) carried 3 virulence genes. In two isolates (2.4 %) 6 virulence genes were identified. None of isolates carried 5 or 7 virulence genes. Phenotypically, non of isolates produced gelatinase despite being *gelE*-positive in PCR.

### Antibiotic susceptibility

One (0.82 %) out of the 82 clinical *E. cecorum* was susceptible to 13 antibiotics tested, the rest were resistant to one or more antibiotics (Table 4). All isolates were susceptible to amoxicillin/clavulanic acid (AUG) and penicillin (PG), nitrofurantoin (NI), and linezolid (LZD). The majority of isolates were susceptible to ampicillin (AP), and high level gentamicin (GM) (*n* = 81; 99 %), chloramphenicol (C) (*n* = 79; 96 %), vancomycin (VA) (*n* = 75; 91 %). The lower level susceptibility was to erythromycin (E) (*n* = 42; 51 %), tetracycline (TEC) (*n* = 24; 29 %), teicoplanin (T) and doxycycline (DXT) (*n* = 11; 13 %). Most isolates noted intermediate susceptibility to TEC (*n* = 53; 65 %) with 29 % susceptible and 6 % resistant. None of clinical *E. cecorum* isolates was susceptible to enrofloxacin (71 resistant isolates). A high percentage of antimicrobial resistance was also observed to teicoplanin (T) (*n* = 70; 85 %), and doxycycline (DXT) (*n* = 68; 83 %). Linezolid resistance among *E. cecorum* isolates was not found. Of the vancomycin resistance genes tested by PCR, *vanA* gene was present in one strain, *vanB* gene was not detected.

**Table 4:** Antibiotics resistance patterns of *E. cecorum* strains isolated from clinical cases of different bird species

Antibiotics (*n*, number of antibiotics)	% (*n*) of resistant isolates
ENF/E/T/DXT/TEC (5)	2.5 (2)
ENF/E/T/DXT (4)	33.3 (27)
ENF/T/DXT/TEC (4)	1.2 (1)
ENF/T/DXT/AP (4)	1.2 (1)
ENF/T/DXT (3)	33.3 (27)
T/E/DXT (3)	4.9 (4)
ENF/E/T (3)	2.5 (2)
ENF/E/GM (3)	1.2 (1)
ENF/DXT/VA (3)	1.2 (1)
T/DXT (2)	6.2 (5)
ENF/E (2)	2.5 (2)
ENF/TEC (2)	1.2 (1)
T/TEC (2)	1.2 (1)
ENF (1)	7.4 (6)
0	1.2 (1)

Ampicillin (AP 10 μg), enrofloxacin (ENF 5 μg), tetracycline (TEC 30 μg), Doxycycline (DXT 30 μg), erythromycin (E 15 μg), teicoplanin (T 30 μg), Vancomycin (VA 30 μg), high level gentamicin (GM 120 μg)

### Molecular identification

**Fig. 2:**
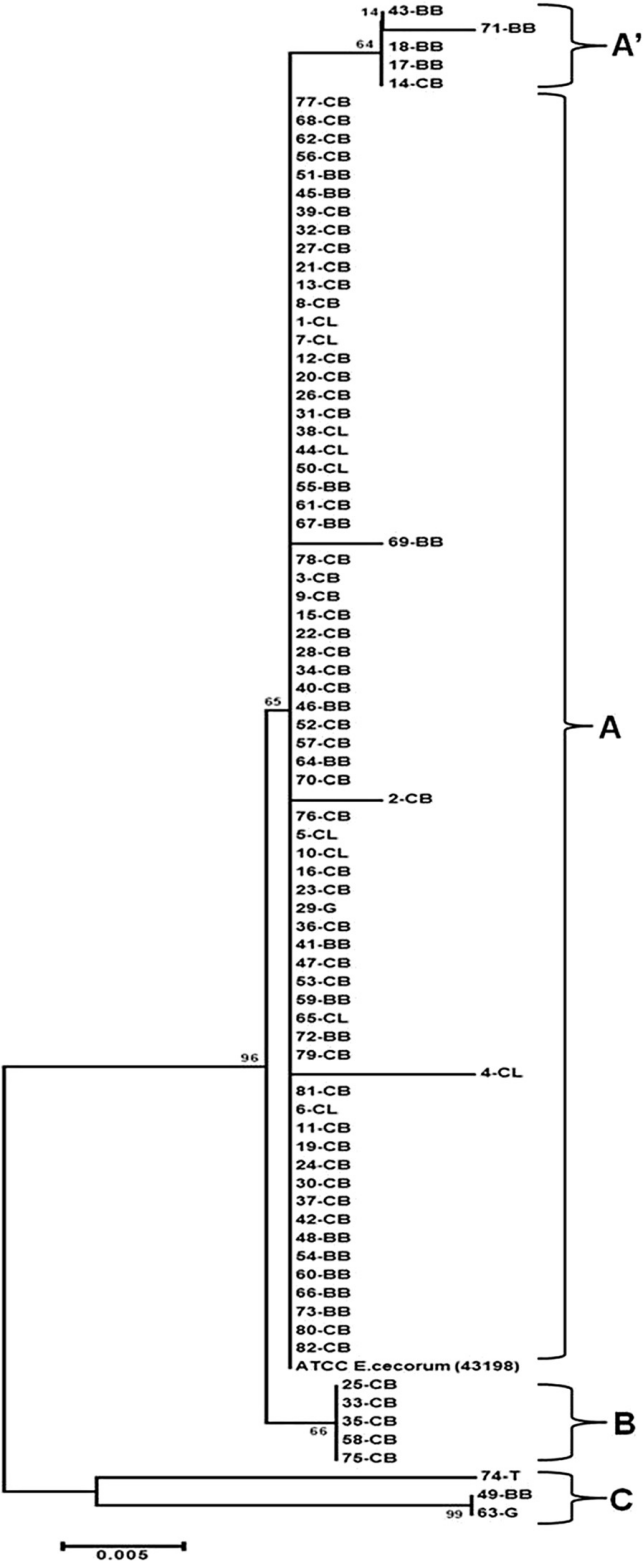
Phylogenetic tree constructed using the Neighbor-Joining algorithm to evaluate the distance between partial *sodA* gene sequences of 82 clinical *Enterococcus cecorum* poultry-origin strains and *E. cecorum* reference strain (ATCC 43198). The bootstrap values (1000 replicates) are reported as percentage greater than 60 %. The evolutionary distances were computed using the Kimura 2-parameter method and are in the units of the number of base substitutions per site. Evolutionary analyses were conducted in MEGA6

The obtained sequences *sod*A gene fragment showed similarity to *E. cecorum* (BLAST database) and allowed for identification strains. Dendrogram showed the genetic similarity between reference strain of *E. cecorum* and clinical isolates based on the *sod*A gene sequences (Fig. 2). Phylogenetic analysis supported the separation of clinical isolates into three main groups (A, B, C). Genetic distances between groups ranged from 0.00 to 0.04 (Table 5). The group A comprised 69 strains (CB *n* = 43, BB *n* = 15, CL *n* = 10, G *n* = 1) and had one subgroup (A’) with 5 strains (BB *n* = 4, CB *n* = 1). Five CB isolates were clustered together in the group B, and three isolates (BB, G, T) in the group C (all isolates from 2014). Among all groups, the group C revealed the highest values of genetic distance with B group (0.04) and with reference strain (0.03).

**Table 5:** Kimura 2-parameter genetic distances between groups of clinical *E. cecorum* (A, A’, B, C) and reference strain (ATCC 43198)

	1	2	3	4	5
1. A					
2.A’	0.00				
3.B	0.00	0.01			
4.C	0.03	0.04	0.03		
5.ATCC (43198)	0.00	0.00	0.00	0.03	

### PFGE

**Fig. 3:**
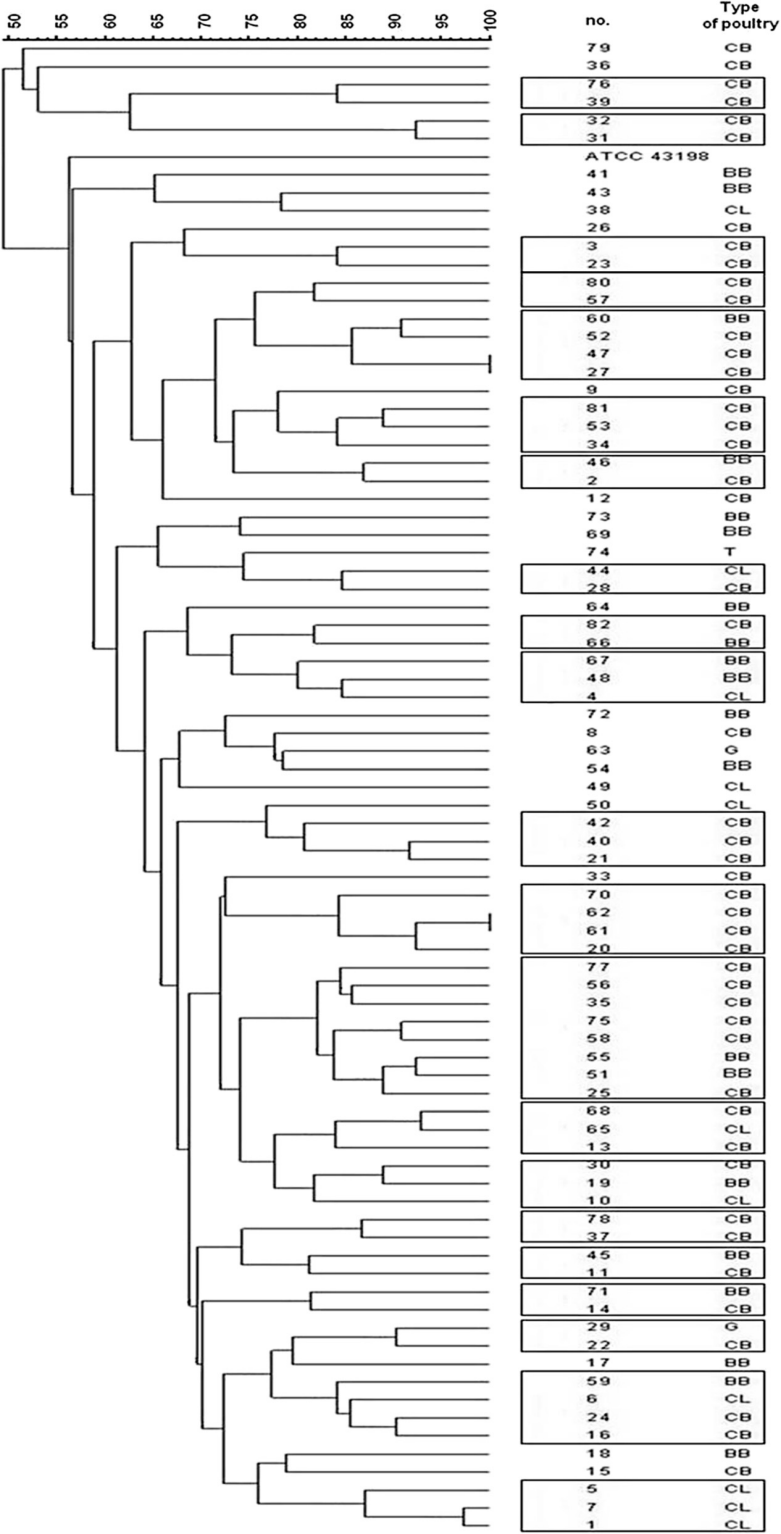
Results of pulsed field gel electrophoresis (PFGE) examination of *Enterococcus cecorum* clinical isolates. Dendrogram based on Dice coefficient with 1 % position tolerance. Cut-off value of 80 % similarity was used to assign the pulsotypes

The PFGE analysis (based on >80 % similarity index) of 82 clinical *E. cecorum* isolates exhibited 21 pulsotypes (A-U) with 60 strains (41 CB, 10 BB, 8 CL, 1 G) (Table 6, Fig. 3). The highest degree of band similarity (>90 %) was demonstrated in pulsotype B (with two CB isolates) and in pulsotype S (with G and CB isolate). Pulsotype M was the predominant type, and included 8 isolates (8/60, 13.3 %), then E, L, T pulsotypes (each included 4 isolates). However, 11 of the 21 pulsotypes included only 2 isolates. Twenty isolates (20/60, 33.3 %) representing CB flocks (20/41; 48.8 %), were distributed among 8 pulsotypes: A, B, C, D, P (each 3.33 %), F, K (each 5 %), L (6.6 %). The majority of BB isolates (7/10, 70 %) were clustered with CB isolates (13) in distinct 6 pulsotypes (E, G, I, M, Q, R). Among isolates representing CL flocks (8/60, 13.3 %), three of these (3/8, 37.5 %) were clustered in one profile (L). Three pulsotypes (H, N, O) were created by clustering both CB (5) and CL (3) isolates. Generally, no clear temporal and geographical clustering was visible, but with the exceptions of 7 pulsotypes (A, D, I, L, M, P, U).

**Table 6:** Twenty one PFGE profiles (A-U) of clinical isolates *E. cecorum* derived from poultry in Poland between 2011-2014

Pulsotype	Poultry type	No. of strain	Year	Poland’s voivodeship	Number of isolates of each pulsotype	% Similarity (>80 %)
A	CB	76	2014	Greater Poland	2	84.2
	CB	39	2014	Greater Poland		
B	CB	32	2014	Greater Poland	2	92.3
	CB	31	2013	Świętokrzyskie		
C	CB	3	2011	Greater Poland	2	84.2
	CB	23	2012	Silesian		
D	CB	80	2014	Masovian	2	81.8
	CB	57	2014	Masovian		
E	BB	60	2014	Warmian-Masurian	4	85.7
	CB	52	2014	Pomeranian		
	CB	47	2014	Greater Poland		
	CB	27	2013	Greater Poland		
F	CB	81	2014	Masovian	3	84.2
	CB	53	2014	Pomeranian		
	CB	34	2014	Greater Poland		
G	BB	46	2014	Masovian	2	87.0
	CB	2	2011	Greater Poland		
H	CL	44	2014	Podlaskie	2	84.6
	CB	28	2013	Greater Poland		
I	CB	82	2014	Masovian	2	81.2
	BB	66	2014	Opolskie		
J	BB	67	2014	Masovian	3	80.1
	BB	48	2014	Masovian		
	CL	4	2011	Greater Poland		
K	CB	42	2014	Pomeranian	3	80.8
	CB	40	2014	Pomeranian		
	CB	21	2012	Warmian-Masurian		
L	CB	70	2014	Masovian	4	84.3
	CB	62	2014	Masovian		
	CB	61	2014	Masovian		
	CB	20	2014	Masovian		
M	CB	77	2014	Kuyavian-Pomeranian	8	82.1
	CB	56	2014	Greater Poland		
	CB	35	2014	Greater Poland		
	CB	25	2014	Pomeranian		
	CB	58	2014	Pomeranian		
	CB	75	2014	Lodzkie		
	BB	51	2014	West Pomeranian		
	BB	55	2014	West Pomeranian		
N	CB	68	2014	Masovian	3	84.0
	CL	65	2014	Masovian		
	CB	13	2011	Greater Poland		
O	CB	30	2013	Masovian	3	81.8
	CB	19	2011	Świętokrzyskie		
	CL	10	2011	Greater Poland		
P	CB	78	2014	Greater Poland	2	86.7
	CB	37	2014	Greater Poland		
Q	BB	45	2014	West Pomeranian	2	81.3
	CB	11	2011	Greater Poland		
R	BB	71	2014	Masovian	2	81.5
	CB	14	2011	Greater Poland		
S	G	29	2013	Greater Poland	2	90.3
	CB	22	2012	Pomeranian		
T	BB	59	2014	Warmian-Masurian	4	84.1
	CL	6	2011	Greater Poland		
	CB	16	2011	Greater Poland		
	CB	24	2012	Masovian		
U	CL	1	2012	Greater Poland	3	87.0
	CL	5	2012	Greater Poland		
	CL	7	2011	Greater Poland

## Discussion

In order to characterize clinical *E. cecorum*, we investigated 82 strains isolated from clinical samples originated from different poultry flocks (1 isolate per flock). Our observations were consistent with reports on a succession of disease outbreaks in broiler flocks raised in the intensive farming systems [9]. Previously, clinical *E. cecorum* was not described in commercial chicken layers or geese flocks. We found that the problem may affect hens or other bird species than chicken. Our results were consistent with the literature in regarding on certain characteristics traditionally considered to be typical for the genus *Enterococcus* or *E. cecorum* including intestinal isolates of poultry origin [28, 29]. According to the literature, *E. cecorum* are often NaCl sensitive [17, 30], and intestinal *E. cecorum* of poultry may be also NaCl-resistant [28]. In our study, clinical isolates appeared to be less salt-tolerant, however no complete inhibition of growth was observed. Authors suggested possible higher ability to survive clinical *E. cecorum* in saline environment or even higher resistance to chlorine-based disinfectants.

Previous research demonstrated no growth of poultry cecal *E. cecorum* on Slanetz medium, and on KAA agar [1], while clinical strains showed variable growth on these media. The growth was clearly more abundant on bile esculine azide agar than on esculin azide agar with kanamycin. Based on results, we suggested that complete growth inhibition on a solid medium supplemented with tergitol or with potassium tellurite may be used in identification of this enterococcal species. According to the literature, *Enterococcus* species are able to survive a range of stresses and hostile environments [31], but *E. cecorum* was described as unable to grow at 10 °C or survive 30 min at 60 °C [5, 29]. In contrast to above authors, clinical isolates were able to grow at low temperatures (4 °C, 10 °C) and some of them might survive even longer heating at 60 °C for 1 h and even 70 °C for 30 min. The results may indicate to the possibly longer survival *E. cecorum* at more extreme temperatures in the poultry house environment.

We confirmed the efficacy of two biochemical systems for identification poultry-origin *E. cecorum* strains. Instead of doubtful or unacceptable profile in API, all strains were properly recognized by *sod*A gene sequencing. We found, that almost all clinical strains gave positive reactions in 10 biochemical tests, and negative in 5 tests (API). Similar results were reported for other *E. cecorum* including commensal or reference strains with some exceptions [1, 17, 28, 32]. We observed that all of strains were able to metabolise α-cyclodextrin. Makrai et al. [10] observed differences among clinical isolates in metabolism of both α- and β-cyclodextrin. We noted relatively high positive reactions for βGAL, βGAR, VP, opposed to reference *E. cecorum* strain and despite the discrepancies in the literature [17]. In contrast to other studies [2, 17], some clinical *E. cecorum* showed ability to produce urease, β-mannosidase, and metabolize glycogen. On the other hand, results for β-mannosidase, glycogen, mannitol were lower for clinical isolates than reported for standard strains. Our results were consistent with Borst et al. [33] who noted that pathogenic *E. cecorum* isolates are more deficient in mannitol metabolism. Recently molecular aspects for the defect mannitol metabolism in pathogenic strains were investigated [34].

Based on comparative analysis of our results with study of Makrai et al. [10], it could be assumed, that all clinical *E. cecorum* may metabolise 18 carbon sources (adenosine, arbutin, D-cellobiose, dextrin, D-fructose, D-mannose, D-psicose, D-raffinose, D-trehalose, gentiobiose, maltose, maltotriose, N-acetyl-D-glucosamine, N-acetyl-β-D-mannosamine, pyruvic acid methyl ester, salicin, sucrose, α-D-glucose). Similarly to above mentioned authors, clinical isolates may give differences in 3 tests: α-D-lactose, D-ribose, 2-deoxy adenosine.

Recently several potential mediators of virulence were found in pathogenic *E. cecorum* isolated from chickens in the southeast US. These virulence determinants conserved in pathogenic EC were found to be similar to those utilized by other medically important enterococci [33]. In the present study, only 32.9 % clinical *E. cecorum* strains contained one or more virulence genes. *E. cecorum* from chicken flocks contained mainly *asa1*/*gel*E/*ace* genes. The pathogenicity of *E. cecorum* may be associated with other species-specific virulence factors. Similar observations were presented by Jackson et al. [35] who detected only few virulence genes among US *E. cecorum* isolates, and the incidences of virulence determinants tested were lower than ours. In our study the most of positive isolates contained two *asa1*/*gelE* or four *asa1/gelE*/*efaA*/*ace* virulence genes. We speculated about possible linkage between *asa1*/*gelE* (74 % of all virulence positive isolates) or *efaA*/*ace* (33.3 % of all virulence positive isolates) in clinical *E. cecorum*. It may have impact on pathogenesis and clinical course of infection.

Because none of the investigated strains harbored *hyl* gene, we suggest that this virulence determinant may be not widespread among clinical isolates. Our results were consistent with other authors who described the lack of *hyl* in *E. cecorum* from poultry carcass rinsates, diseased chickens [35] and pigeons [5]. According to the literature, hyaluronidase is a degradative enzyme that is associated with tissue damage. Among *Enteroccocus* species the *hyl* gene has been reported more often in ampicillin-resistant VRE *E. faecium* isolates [36]. We suggest that *hyl* is not specific for *E. cecorum* and could has minor role in pathogenicity of *E. cecorum,* however more studies are needed to elucidate this aspect.

The present study showed lack of correlation between the presence of *gelE* gene and its expression. The literature provide no data in regard this aspect on *E. cecorum*, however similar observations are available for *E. faecalis* [37].

Generally, pathogenic isolates from poultry were found to be significantly more drug resistant than commensal strains [33]. In the present study almost all of clinical isolates showed high level of antibiotic resistance and 91.5 % of them showed multidrug resistance (resistance to ≥ 2 antimicrobials). Other authors identified lower multidrug resistance in *E. cecorum* from carcass rinsates and diseased poultry, however the panel of used antibicrobials were not completely the same [35]. Affected flocks were treated against *E. cecorum* usually with amoxicillin, doxycycline or enrofloxacin. All of the above antibiotics were tested in this study. Similarly to other authors, the overwhelming majority of the isolates were susceptible to penicillin, which appear to be drug of choice [4, 5, 7, 10, 14, 15]. However, the majority of *E. cecorum* was resistant to enrofloxacin > teicoplanin > doxycycline > erythromycin. Our results were opposed to clinical *E. cecorum* from other countries, in which sensitivity to enrofloxacin (in Germany, Holland, Hungary, South Africa), doxycycline (in Germany, Hungary) and macrolides (in Belgium, Germany) were identified [4, 5, 7, 10, 14, 15]. Similarly to the isolates from Canada, USA, Holland and Belgium, clinical *E. cecorum* from Poland showed the increased resistance to tetracycline or erythromycin (macrolides) [4, 7, 27, 33, 35]. This antimicrobial resistance pattern may be common and characteristic for pathogenic *E. cecorum.* The presented study indicated on the presence clinical *E. cecorum* (1.2 %) with the resistance to vancomycin (VRE) and to high level gentamicin (HLGR). Similarly to Jackson et al. [35], we found out that none of the isolates were resistant to linezolid. According to the literature, enterococci have both an intrinsic and acquired resistance to antibiotics which complicate treatment of infections. The acquired resistance includes resistance to i.a. chloramphenicol, tetracyclines, fluoroquinolones, aminoglycosides (high levels), and vancomycin. Enterococci have demonstrated a huge potential for acquiring and disseminating resistant genes. We found, that the high level of the resistance to enrofloxacin, doxycycline, tetracycline in *E. cecorum* isolates is probably related to the wide use of these antibiotics in poultry production. In previous years erythromycin was also commonly applied in the therapy of poultry. Other authors confirmed the presence of resistance genes (including *van* genes) among *E. cecorum* from broilers or retail chicken meat [38, 39]. We suggest, that poultry may play an important role as reservoirs of antibiotic resistant *E. cecorum* in the environment. However, further studies are needed to investigate the resistance genes in clinical isolates.

In the present study *sod*A gene fragment was successfully used to confirm phenotypic identification of *E. cecorum,* however it was not sufficiently discriminative to differentiate them from each other. In the collection it was possible to distinguish for three phylogenetic groups and one subgroup. The strains from group B showed the same type of production (CB), year of isolation, virulence determinants and multidrug resistance pattern, but different geographical origin; 80 % of them belong to pulsotype M. The strains of group C shared only the same year of isolation, virulence and multidrug resistance patterns. The low genetic distance (based on *sod*A gene sequences) indicated on the very close genetic relationships between clinical *E. cecorum*. No clear genetic differences were observed between clinical strains and reference strain.

Recent data indicated that pathogenic *E. cecorum* from the southeast US were clonal, however comparative genomic analysis revealed fundamental differences in their genomes [34]. According to the previous report, isolates recovered from spinal abscesses were highly similar and could be detected by using PFGE [33]. In our study, PFGE results showed the genetic heterogeneity between clinical *E. cecorum* isolates, that is consistent with the other studies [18]. Therefore, the usage of PFGE in distinguishing pathogenic strains may be difficult and limited. This genetic diversity was seen between poultry flocks, however some clustering was visible in relation of type of production (CB, CL). Moreover, some temporal and geographical clustering was visible. Many CB isolates from the same year and geographical origin were clustered together (pulsotype A, D, L, P) indicating their close genetic relationship*.* Some CL isolates from the same location but different years were grouped into a single pulsotype (U) indicating on the possible horizontal transmission among CL flocks in this area. We found that CB and BB isolates from the same year which were clustered together into separate pulsotypes (I, M). Based on the relatively close relationship between isolates from geese and chicken flocks, it could be assumed that isolates from the single clonal lineage may cause outbreaks in different bird species. The results may suggest the transmission of potential disease-causing *E. cecorum* between flocks.

## Conclusions

These data indicate that several, widely distributed pathogenic *E. cecorum* clones seemed to be responsible for infection cases found in different poultry types. The isolates causing infection in different CB flock in the same year and region may be somewhat genetically distinct from each other and from those that cause disease in CL or BB flocks in the same year and region. Phenotypically, clinical isolates were generally found to be very similar, however some properties or characteristics described in some isolates were not found in others. The study presented here is the first in Poland as well as one of the few in Europe which provides phenotypic and genotypic characterization of *E. cecorum* isolates associated with disease outbreaks in poultry flocks. Further research needs to focus on finding new virulence determinants of *E. cecorum* and recognition of transmission routes.

## Abbreviations

BB, broiler breeder flocks; CA, Columbia agar with 5 % sheep blood; CB, chicken broiler flocks (commercial broilers); CL, commercial layer flocks; *E. cecorum, Enterococcus cecorum;* ES, enterococcal spondylitis; G, geese flocks; HLGR, high level gentamicin resistance; PFGE, Pulsed Field Gel Electrophoresis; T, turkey flock; VRE Vancomycin-Resistant Enterococcus

## Additional files

Additional file 1: Alignment of partial *sod*A gene sequences from *E. cecorum* isolates and reference strain (ATCC 43198). Nucleotide differences are specified by the nucleotide, while dot represented no nucleotide changing. (TIF 12403 kb) Additional file 2: Alignment of partial 16S rRNA gene sequences from *E. cecorum* isolates and reference strain (ATCC 43198). Nucleotide differences are specified by the nucleotide, while dot represented no nucleotide changing. (TIF 60654 kb)
