# Supplementary figures and images for: Phenotypic and genotypic characterization of Enterococcus cecorum strains associated with infections in poultry
Source: BMC Vet Res. 2016 Jun 27;12:129. doi: 10.1186/s12917-016-0761-1 (PMC4924287; doi:10.1186/s12917-016-0761-1)

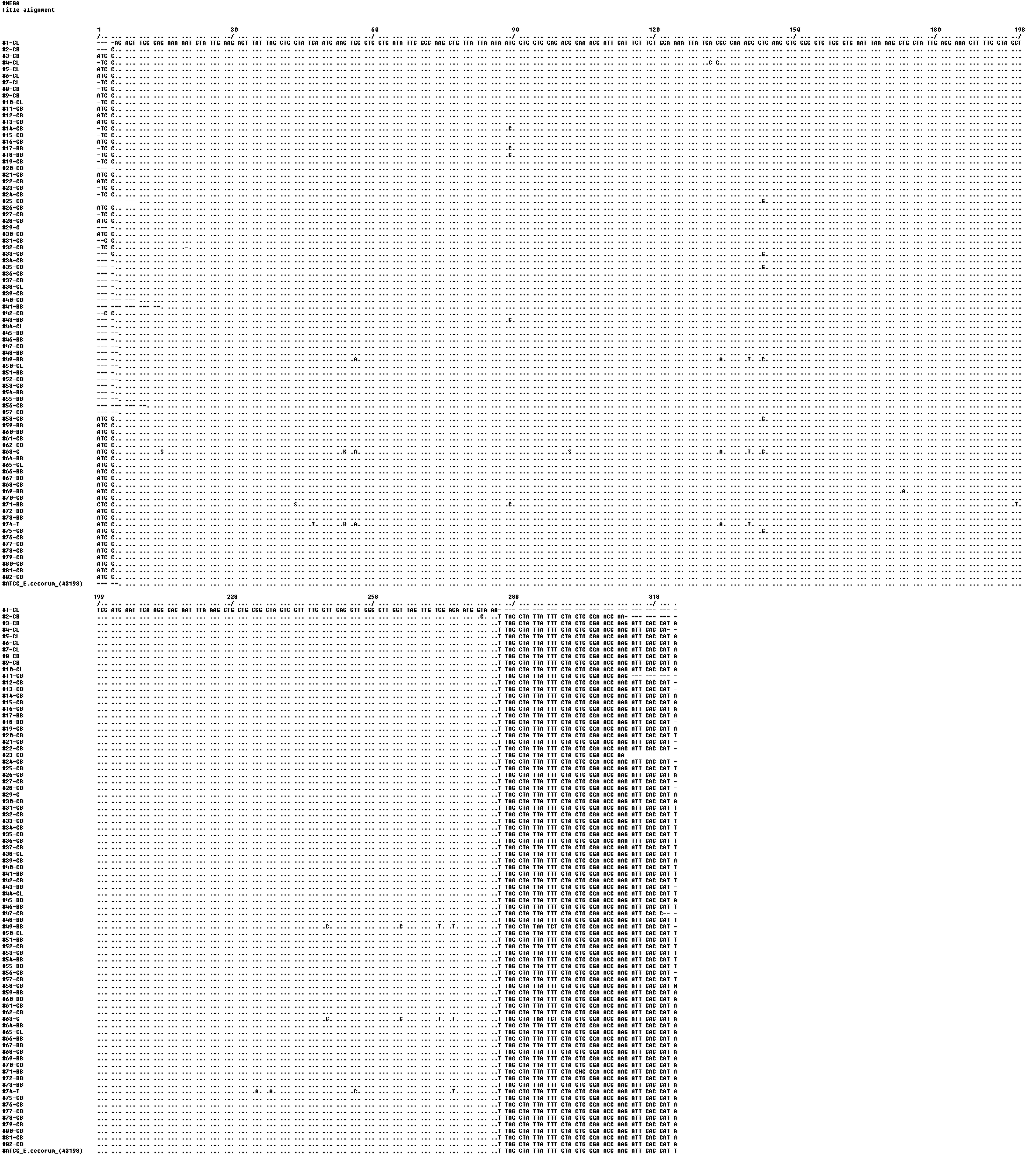

Supplement: Additional file 1: — Alignment of partial sodA gene sequences from E. cecorum isolates and reference strain (ATCC 43198). Nucleotide differences are specified by the nucleotide, while dot represented no nucleotide changing. (TIF 12403 kb) [file 12917_2016_761_MOESM1_ESM.tif]
